# Psychometric properties of two measures of psychological well-being in adult growth hormone deficiency

**DOI:** 10.1186/1477-7525-4-16

**Published:** 2006-03-22

**Authors:** Carolyn V McMillan, Clare Bradley, James Gibney, David L Russell-Jones, Peter H Sönksen

**Affiliations:** 1 Department of Psychology, Royal Holloway, University of London, Egham, Surrey, TW20 0EX, UK; 2 Department of Endocrinology, Adelaide & Meath Hospitals, incorporating the National Children's Hospital, Tallaght, Dublin 24, Eire, UK; 3 Department of Diabetes and Endocrinology, Royal Surrey County Hospital, Guildford, UK; 4 GKT School of Medicine, St Thomas' Campus, London, UK

## Abstract

**Background:**

Psychometric properties of two measures of psychological well-being were evaluated for adults with growth hormone deficiency (GHD): the General Well-being Index, (GWBI) – British version of the Psychological General Well-being Index, and the 12-item Well-being Questionnaire (W-BQ12).

**Methods:**

Reliability, structure and other aspects of validity were investigated in a cross-sectional study of 157 adults with treated or untreated GHD, and sensitivity to change in a randomised placebo-controlled study of three months' growth hormone (GH) withdrawal from 12 of 21 GH-treated adults.

**Results:**

Very high completion rates were evidence that both questionnaires were acceptable to respondents. Factor analyses did not indicate the existence of useful GWBI subscales, but confirmed the validity of calculating a GWBI Total score. However, very high internal consistency reliability (Cronbach's alpha = 0.96, N = 152), probably indicated some item redundancy in the 22-item GWBI. On the other hand, factor analyses confirmed the validity of the three W-BQ12 subscales of Negative Well-being, Energy, and Positive Well-being, each having excellent internal reliability (alphas of 0.86, 0.86 and 0.88, respectively, N from 152 to 154). There was no sign of item redundancy in the highly acceptable Cronbach's alpha of 0.93 (N = 148) for the whole W-BQ12 scale. Whilst neither questionnaire found significant differences between GH-treated and non-GH-treated patients, there were correlations (for GH-treated patients) with duration of GH treatment for GWBI Total (r = -0.36, p = 0.001, N = 85), W-BQ12 Total (r = 0.35, p = 0.001, N = 88) and for all W-BQ12 subscales: thus the longer the duration of GH treatment (ranging from 0.5 to 10 years), the better the well-being. Both questionnaires found that men had significantly better overall well-being than women. The W-BQ12 was more sensitive to change than the GWBI in the GH-Withdrawal study. A significant between-group difference in change in W-BQ12 Energy scores was found [t(18) = 3.25, p = 0.004, 2-tailed]: patients withdrawn from GH had reduced energy at end-point. The GWBI found no significant change.

**Conclusion:**

The W-BQ12 is recommended in preference to the GWBI to measure well-being in adult GHD: it is considerably shorter, has three useful subscales, and has greater sensitivity to change.

## Background

The physical symptoms of adult growth hormone deficiency (GHD) include abnormal body composition with reduced lean body mass and increased central adiposity; reduced muscle strength and exercise performance [1]. However, adults with untreated GHD report symptoms of reduced psychological well-being including low energy, tiredness, irritability [2], anxiety, depression and mood swings [3] and it has been said that "psychological aspects may be at least as, if not more, important" than physiological [4]. To measure the effects of growth hormone (GH) treatment sensitive measures of patient-reported outcomes, such as quality of life (QoL), health status and psychological well-being, are also required in addition to measuring physiological outcomes. Condition-specific QoL measures in adult GHD exist [5,6], but QoL is not equivalent to health status or well-being, although often reported as such [7].

The questionnaire most commonly employed to measure psychological well-being in adult GHD has been the generic 22-item Psychological General Well-being Index (PGWB) [8], with six subscales (Anxiety, Depression, General Health, Positive Well-being, Self-control and Vitality), and a Total score. Results of randomised placebo-controlled trials providing GH treatment to adults with GHD have been mixed. In some studies the PGWB found no significant change [9-11], but others found, after six months' GH-treatment, significant improvement in Depression [12]; Vitality and Total well-being [13]; General Health, Positive Well-being, Vitality and Total well-being [14]. Longer-term open-label studies of GH treatment have found improvements in most or all PGWB variables [15-17]. Those who continued with GH therapy over 9 years had significant increases in Vitality, but those who discontinued GH had significant decreases in General Health over the same period [18]. Some were British studies [9,12,14,15,18] but used the PGWB, although this American questionnaire does not appear to have been validated either for use in the UK or in adult GHD. However, a British version of the PGWB exists: the General Well-being Index (GWBI) [19]. This is very similar to the PGWB having only some minor differences in vocabulary (e.g. *blue *becomes *sad *on the British version), five response categories rather than the six, and in question order.

Validation of the GWBI, in two samples of British patients with depression, indicated construct validity [19,20] and high internal consistency reliability for the whole scale (in the range 0.92 – 0.96) [19]. Ability to discriminate between subgroups in a primary care setting has also been demonstrated [21]. Subscales were not recommended owing to their high inter-correlations and lack of adequate internal consistency (alpha scores were not supplied) [20], although alpha scores for PGWB subscales have been reported (ranging from 0.72 (Self-control) to 0.88 (Anxiety) [8]. However, more recently Gaston and Vogl investigated the psychometric properties in an Australian non-clinical population and found three significant factors [22], rather than the six factors that might have been expected given that the GWBI is so similar to the six-subscale PGWB.

The Well-being Questionnaire (W-BQ) [23] is another generic measure of psychological well-being. The 12-item version, the W-BQ12, is derived from the longer W-BQ22 [23,24], and which has been used in a number of studies to evaluate the effects of new treatments and interventions in diabetes [23-25], a condition for which it has good internal consistency and validity [25-27]. The W-BQ12, however, poses less respondent burden than the W-BQ22, and redresses an imbalance between numbers of positively worded and negatively worded items in the longer questionnaire [28]. The W-BQ12 has been translated into several languages, and psychometric evaluations of these and the original English have confirmed its structure and reliability for people with diabetes [25-27] and it also has good psychometric properties for people with macular disease [29].

The two studies described here presented the opportunity to evaluate the psychometric properties of both the GWBI and W-BQ12 in a sample of adults with GHD at a London hospital. The first study was a cross-sectional survey of 157 adults with severe GHD, GH-treated and non-GH-treated, to investigate reliability, factor structure, construct and concurrent validity of the questionnaires. Sensitivity to change was investigated in a randomised placebo-controlled study of three months' withdrawal of GH treatment from 12 of 21 GH-treated adults, where nine continued with GH.

## Methods

### The questionnaires

#### GWBI

The GWBI has 22 questions each with five response options (scoring from 1 to 5), but worded differently for each question, to define the intended meaning (e.g. *During the past two weeks, have you been waking up feeling fresh and rested? Every day – Most days – Less than half the time – Not often – Not at all*). Half the items are positively worded, and half negatively worded. There are no recommended subscales. Scoring: The GWBI Total score is the sum of all 22 items (after reversing the negatively-worded items), and ranges 22 -110. Higher scores indicate worse well-being.

#### W-BQ12

The 12 items of the W-BQ12 are simple statements (e.g. *I feel afraid for no reason at all*), and have four response options from 0 ('*not at all*') to 3 ('*all the time*'), identical in all 12 items. There are three subscales of four items each: Negative Well-being (all negatively worded items), Energy (two positively worded and two negatively worded items) and Positive Well-being (all positively worded). Scoring: Subscale scores range from 0 – 12 (higher scores indicating increased mood of the subscale label). The W-BQ12 General Well-being total score is the sum of all 12 items (after reversing the Negative Well-being item scores), and ranges from 0 – 36 (higher scores indicating better well-being).

#### Other questionnaires

Other questionnaires were also completed in these studies including the Nottingham Health Profile [30], the Short-form 36 (SF-36) [31], and a new hormone deficiency-specific individualised quality of life questionnaire (HDQoL) [5], the full results for which have been reported previously [32-34].

### Study 1: The cross-sectional survey

#### Recruitment procedures

Recruitment procedures have been fully described elsewhere [32], but in brief they were as follows. Participating patients had been diagnosed severely GH deficient as determined by an Insulin Tolerance or Pituitary Function Test in which insulin reduced blood glucose to ≤ 2.5 mmol/L with peak GH concentration ≤ 10 mU/L. Patients had either received GH-replacement therapy for at least six months immediately prior to the study or had not received GH treatment in the previous six months; were aged between 18–70 years; had received appropriate adrenal, thyroid and gonadal hormone replacement therapy as required by their hormonal condition, for at least 12 months prior to the study. Patients might have had adult or childhood onset of GHD. Exclusion criteria were diabetes mellitus, active malignancy or pregnancy.

### Statistical analyses

#### Normality issues

Normality of distributions was investigated through standardised z (skew) values [35]. Some questionnaire item distributions were skewed, and finding transformations for these variables that did not adversely affect normal distributions of other items in the same questionnaire proved difficult. Item data were not transformed to normality, thereby sacrificing some of the accuracy of reliability and factor analyses for the convenience of having interpretability of original units. The assumption was made that if reliability were high, the factor analysis robust, and the number of respondents sufficiently high, then a degree of non-normality was acceptable. In sub-group analyses, Mann-Whitney tests were performed on skewed variables, and t-tests on normal data.

#### Internal consistency reliability and factor structure

Cronbach's alpha coefficient [36] was determined with an acceptable minimum alpha being taken as 0.7 to 0.8, depending on the number of items in a scale [37], noting that some consider 0.9 as the minimum for measures of differences between individuals [38]. Acceptable corrected item-total correlations are those >0.2 [39]. Factor structure was explored using Principal Components extraction with Varimax rotation. Salient loadings were taken as ≥ 0.4, higher than the recommended minimum 0.3 [40], erring on the side of caution in an effort to reduce the risk of spurious loadings that owed their origin to non-normality of item distributions and to avoid multiple loadings.

#### Subgroup differences and 'familywise' error in multiple tests

The questionnaires' sensitivity to some subgroup differences was investigated (GH-treatment groups, and sex). The Holm's sequential Bonferroni procedure for multiple tests [41] was adopted in large correlation matrices and for the W-BQ12 and its subscales.

#### Concurrent validity

Correlations were undertaken with Mental Health, Vitality and Physical Functioning subscales of the widely used SF-36 health status measure also completed in this study [32]. SF-36 subscale scores range from 0 to100, (higher scores indicating better functioning). Correlations >0.7 indicated adequate convergence [42].

Means are reported as mean (standard deviation).

### Study 2: Prospective study of GH-withdrawal

Preliminary sensitivity to change was assessed in a small randomised, double-blind, placebo-controlled study where severely GH-deficient patients were allocated to placebo or continued treatment with GH for a period of three months. This study has been fully described elsewhere [33]. The GWBI and W-BQ-12 (in a battery of several questionnaires) were completed at baseline and end-point. There was a general clinical expectation of deterioration in physiological factors during the study period for those withdrawn from GH treatment and that this might be accompanied by reduced psychological well-being.

The Guy's and St Thomas' Hospital Trust Ethics Committee gave approval for both studies and patients gave written informed consent before participating. Research was carried out in compliance with the Helsinki Declaration

## Results

### Cross-sectional study

#### The patient sample

Of 219 questionnaires distributed, 163 were returned (74% response rate), but six patients did not meet all inclusion criteria, leaving 157 data sets, (91 GH-treated and 66 non-GH-treated patients). Most patients (96%) had multiple pituitary hormone deficiencies including GHD; the remainder had isolated GHD. (See Table 1 for sample characteristics).

**Table 1:** Characteristics of the 157 patients in the cross-sectional survey

	**GH treatment** **(Maximum N = 91)**	**No GH treatment** **(Maximum N = 66)**
Women	51	33
Men	40	33
Childhood onset of GHD	21	9
Adult onset of GHD	70	57
Isolated GHD	5	1
Multiple pituitary hormone deficiencies	86	65
Mean age (SD) in years	47.1 (12.59)	51.32 (12.41)
[range]	[23.75–70.92]	[23.83–70.92]
Mean duration GHD (SD) in years (adult onset patients)	13.02 (6.78)	13.18 (7.84)
Mean duration GH Treatment (SD) in years	3.6 (2.39)	-
[range]	[0.5–10.17]	-
BMI (kg/m^2^) (SD)	27.16 (5.54)	27.99 (5.25)
Height (cms) (SD)	167.5 (10.53)	168.7 (10.6)

#### Completion rates

The completion rates for the GWBI and W-BQ12 were very high (99.1% and 99.0% respectively) indicating excellent acceptability to respondents.

### Reliability and factor analyses

#### GWBI

Unforced factor analysis of the whole scale produced three factors with eigenvalues >1, accounting for 67.7% of the variance (Table 2). These three factors were very similar to those obtained for a non-clinical population where the factors were described as: Factor 1 ('general mood/affect'), Factor 2 ('life satisfaction/vitality') and Factor 3 ('poor physical health/somatic complaints') [22]. The main differences between the factor loadings obtained on the two studies were for item 14 *tired exhausted*, which loaded highest on Factor 2 (present study) compared to Factor 3 (non-clinical population), and item 6 *happy with personal life *which loaded highest on Factor 1 (present study), compared to Factor 2 (non-clinical sample). There were double loadings >0.4 (present study) across factors for seven items, with two more items potentially double loading (at 0.395).

**Table 2:** GWBI loadings on unforced and forced 1-factor analyses^‡^

**Question**	**Factors in an unforced analysis**			**Forced 1-factor**
	**1**	**2**	**3**	

Q1 *feel in general*	**.586**	*.532*	.222	**.821**
Q2 bothered by nerves*	**.669**	.136	.104	**.588**
Q3 *in firm control*	**.678**	*.459*	.026	**.772**
Q4 sad hopeless*	**.644**	.373	.114	**.724**
Q5 under stress*	**.638**	.066	.395	**.620**
Q6 *happy with personal life*	**.550**	*.486*	.146	**.740**
Q7 losing control*	**.711**	.082	.126	**.591**
Q8 anxious upset*	**.782**	.220	.347	**.801**
Q9 *wake up rested*	.284	**.699**	.231	**.718**
Q10 illness pain*	.159	.304	**.861**	**.592**
Q11 *life full of interest*	.316	**.675**	.090	**.678**
Q12 disheartened sad*	**.728**	.382	.308	**.853**
Q13 *stable sure*	**.618**	*.545*	.057	**.795**
Q14 tired exhausted*	.351	**.667**	.374	**.792**
Q15 depressed*	**.744**	.395	.250	**.853**
Q16 tense*	**.686**	.365	.117	**.749**
Q17 *well enough to do things*	.286	**.632**	*.408*	**.737**
Q18 *energy vitality*	.147	**.822**	.275	**.715**
Q19 worries about health*	.289	.376	**.762**	**.695**
Q20 *active vigorous*	.194	**.841**	.265	**.757**
Q21 *cheerful*	**.616**	*.590*	.063	**.825**
Q22 *relaxed*	**.645**	*.521*	.109	**.816**

% of variance	30.8	25.9	11.0	55.1

^‡^Principal Components extraction.

*Negatively worded item scores have been reversed for comparison with factor analyses conducted by Gaston and Vogl [22].

Salient loadings ≥ 0.4. Loadings in **bold **are highest loadings for an item; *italicised *loadings are salient, but not the highest loading.

As the original PGWB has six subscales, and only minor alterations were made when adapting the measure for use in Britain, a forced 6-factor analysis was conducted to investigate support for the original subscales. However, GWBI items did not load separately as intended on the six factors (Table 3). Factor 1 consisted of vitality items, with one general health item (Q17), and Factor 2 consisted of self-control, positive well-being and depression items. Factor 3 was a mix of anxiety, depression and positive well-being items. There was double loading of nine items on more than one factor and, given the lack of any clear pattern of loading, there was no support for a 6-subscale structure. However, the single factor produced in a forced 1-factor analysis of the 22 items accounted for 55.1% of the variance with all items loading satisfactorily ≥ 0.58, (supporting calculation of the GWBI Total score) (Table 2). Note that the loadings in Tables 2 and 3 reflect the fact that scores on negatively worded GWBI items have been reversed to allow for ease of comparison with the results obtained by Gaston and Vogl [22].

**Table 3:** GWBI loadings on forced 6-factor analyses ^‡^

**Question**	**Factors**					
	**1**	**2**	**3**	**4**	**5**	**6**

Q1 *feel in general*	*.424*	**.437**	.282	.386	.216	.203
Q2 bothered by nerves*	.113	.233	.207	.153	.117	**.878**
Q3 *in firm control*	.343	**.447**	.279	.385	.027	.384
Q4 sad hopeless*	.220	**.698**	.100	.390	.179	.146
Q5 under stress*	.063	.216	**.749**	.135	.294	.099
Q6 *happy with personal life*	.298	.225	**.531**	*.524*	.129	-.015
Q7 losing control*	.132	**.807**	.284	-.009	.068	.107
Q8 anxious upset*	.204	*.523*	**.530**	.181	.286	.274
Q9 *wake up rested*	**.686**	.156	.201	.258	.157	.156
Q10 illness pain*	.314	.090	.183	.107	**.855**	.024
Q11 *life full of interest*	.357	.056	.095	**.746**	.210	.166
Q12 disheartened sad*	.267	*.426*	**.500**	.386	.289	.225
Q13 *stable sure*	.340	*.488*	.200	**.539**	.112	.197
Q14 tired exhausted*	**.705**	.175	.357	.183	.254	.124
Q15 depressed*	.321	**.490**	*.412*	.314	.223	.339
Q16 tense*	*.421*	.288	**.649**	.114	-.043	.313
Q17 *well enough to do things*	**.690**	.211	.150	.120	.325	.239
Q18 *energy vitality*	**.828**	.184	.106	.239	.189	-.036
Q19 worries about health*	.350	.196	.168	.191	**.775**	.147
Q20 *active vigorous*	**.799**	.157	.091	.328	.213	.078
Q21 *cheerful*	.369	*.412*	.357	**.592**	.087	.069
Q22 *relaxed*	.377	.193	**.497**	*.474*	.080	.347

% variance	19.9	14.2	13.4	12.8	9.5	7.5

^‡^Principal Components extraction.

*Negatively worded item scores have been reversed for comparison with factor analyses conducted by Gaston and Vogl [22].

Salient loadings ≥ 0.4. Loadings in **bold **are highest loadings for an item; *italicised *loadings are salient, but not the highest loading.

GWBI whole-scale reliability was very high, (Cronbach's alpha 0.959, standardised item alpha 0.96, N = 152), probably indicating some item redundancy. Corrected item-total correlations ranged from 0.55 to 0.83 and were very respectable, no item would increase scale alpha if deleted. Reliability analysis of the three potential subscales (items loading highest on each factor in the unforced analysis, Table 2) indicated that Cronbach's alpha for the 14 items on Factor 1 (general mood/affect) was 0.949 (N = 154), for the six items on Factor 2 (life satisfaction/vitality) was 0.905 (N = 155) and for the two items on Factor 3 (poor physical health/somatic complaints) was 0.874 (N = 154).

Investigating possible item redundancy further, some items are similar in wording e.g. 'fears about health' appears in Q10 (*Have you been bothered by any illness, pains or fears about your health?*) and Q19 (*Have you had any worries or fears about your health?*); 'sad' is found in Q4 (*Have you felt sad, discouraged or hopeless, so much so that you wondered if life was worthwhile?*) and Q12 (*Have you felt disheartened and sad?*). When the unforced factor analysis was conducted with either item Q10 or Q19 deleted from the scale, two factors emerged, with the health item (Q10 or Q19) loading (>0.6) on Factor 2, and the remaining items loading as for the original 22-item scale.

#### W-BQ12

The single factor produced in a forced 1-factor analysis, where all items loaded ≥ ± 0.6, accounted for 56.3% of the variance, confirming the validity of the W-BQ12 General Well-being total score. An unforced factor analysis of the whole scale produced two components with multiple loadings (results not shown). A forced 3-factor analysis (74.6% of the variance) found the four Positive Well-being items and the two positively worded Energy items loading on Factor 1 at >0.4; all four Negative Well-being items loaded on Factor 2 and all four Energy items loaded on Factor 3 with Negative Well-being item 2 (Table 4). There was some double loading found for items 2, 5 and 8. Forced 1-factor analyses of subscales found all items loading >0.8 on their respective subscales, confirming the acceptability of calculating subscale scores.

**Table 4:** W-BQ12 loadings on forced 3-factor and 1-factor analyses ^‡^

**Item (subscale)**	**Factors in forced 3-factor analysis**			**Forced 1-factor analysis**
	**1**	**2**	**3**	

1: crying spells*(Negative Well-being)	-.357	**.753**	.185	**-.732**
2: downhearted* (Negative Well-being)	-.313	**.575**	*.511*	**-.773**
3: afraid no reason* (Negative Well-being)	-.125	**.866**	.127	**-.603**
4: upset panicky* (Negative Well-being)	-.248	**.805**	.213	**-.698**
5: *energetic active *(Energy)	**.691**	-.130	*-.436*	**.763**
6: dull sluggish* (Energy)	-.321	.260	**.825**	**-.772**
7: worn out* (Energy)	-.291	.210	**.862**	**-.744**
8: *wake rested *(Energy)	**.646**	-.135	*-.449*	**.742**
9: *happy *(Positive Well-being)	**.711**	-.325	-.327	**.823**
10: *lived life wanted *(Positive Well-being)	**.769**	-.287	-.176	**.768**
11: *eager tackle tasks *(Positive Well-being)	**.829**	-.172	-.285	**.803**
12: *cope *(Positive Well-being)	**.772**	-.350	-.093	**.761**

% variance	31.37	23.18	19.99	56.3

^‡^Principal Components extraction.

*Negatively worded items, the raw scores of which have not been reversed.

Salient loadings ≥ 0.4. Loadings in **bold **are highest loadings for an item; italicised loadings are salient, but not the highest loading.

A high Cronbach's alpha (0.93) was obtained for the whole scale (N = 148). Corrected item-total correlations were satisfactory (>0.5). The alpha coefficients were very high for each of the 4-item subscales: Negative Well-being (0.86, N = 153), Energy (0.86, N = 152) and Positive Well-being (0.88, N = 154), with corrected item-total correlations >0.65. No item would increase scale or subscale alpha if deleted.

#### Subgroup differences

There were no significant differences in well-being between GH-treated and non-GH-treated patients. Mean GWBI Total of GH-treated patients was 51.2 (S.D. 15.51) and of non-GH-treated patients was 50.89 (17.4) (p = 0.91, *t*-test); mean W-BQ12 General Well-being total of GH-treated patients was 21.84 (S.D. 8.15) and of non-GH-treated patients was 23.18 (8.43) (p = 0.32, *t*-test). Nor were significant differences found between those with childhood onset GHD (N = 29) and adult onset (N = 127). Mean GWBI Total of patients with childhood-onset GHD was 52.9 (S.D. 19.2) and those with adult-onset was 50.67 (15.61) (p = 0.52, *t*-test); mean W-BQ12 General Well-being total of patients with childhood-onset GHD was 20.66 (S.D. 8.77) and of those with adult-onset was 22.8 (8.14) (p = 0.21, *t*-test). Men had significantly better overall well-being than women (GWBI Total and W-BQ12 General Well-being total) and significantly reduced W-BQ Negative Well-being compared with women, (see Table 5).

**Table 5:** Means for men and women in the cross-sectional survey

	**Men**	**Women**	**Significance of differences**
	Mean (SD) [Median] (Minimum N = 71)	Mean (SD) [Median] (Minimum N = 81)	
**GWBI **Total	48.07 (15.27) [47]	53.70 (16.76) [52]	t(150) = -2.15, p = 0.033

**W-BQ12**			
General Well-being total	24.50 (7.86) [25]	20.55 (8.23) [21]	t(154) = 3.05, p = 0.003
Negative Well-being	1.84 (2.32) [1]	3.68 (2.89) [3]	U = 1853.5, p < 0.001, N = 157
Energy	6.87 (3.32) [7]	5.92 (3.20) [6]	n.s (p = 0.07)
Positive Well-being	7.47 (3.16) [8]	6.36 (3.33) [7]	n.s (p = 0.035)*

GWBI: score range 22 – 110 (lower score indicates better well-being).

W-BQ12: subscale score range 0 – 12 (higher scores indicating increased mood of the subscale label); General Well-being total range 0 – 36 (higher score indicating better well-being).

*Not significant after Bonferroni correction (required p value of 0.05/3 = 0.017) applied.

#### Correlations with duration of GH treatment

There were significant negative correlations between duration of GH treatment and GWBI Total (r = -0.36, p = 0.001, N = 85) and W-BQ12 Negative Well-being (rho = -0.37, p < 0.001, N = 88) and significant positive correlations (N = 88) with W-BQ12 Total (r = 0.35, p = 0.001), Positive Well-being (r = 0.33, p = 0.002), and Energy (r = 0.23, p = 0.029). Thus, the longer the duration of GH treatment (ranging from 0.5 to 10 years in this patient sample), the better the well-being.

#### Concurrent validity with SF-36 subscales

GWBI Total correlated strongly and negatively with SF-36 Mental Health (-0.83) and Vitality (-0.82) but had lower correlations with Physical Functioning (-0.47) as might be expected. W-BQ12 General Well-being total correlated strongly with SF-36 Mental Health (0.80). W-BQ12 Energy correlated highly with Vitality (0.80); W-BQ12 Positive Well-being and Negative Well-being correlated moderately highly with SF-36 Mental Health (0.73 and -0.74 respectively), but their correlations with Physical Functioning were lower (-0.31 to 0.49) as expected. Negative correlations were obtained, as expected, where questionnaires are scored in the opposite direction. Note: all correlations were Spearman's rho and significant, p <0.001, 2-tailed, N ranged from 142 to 157.

#### GH-Withdrawal study

The data of 21 patients (age range 25–68 years), all but two with multiple pituitary hormone deficiencies, were available for analysis: 12 placebo-treated (6 men and 6 women) and nine GH-treated (4 men and 5 women). Three months after baseline the serum total Insulin-like Growth Factor-I of placebo-treated patients fell from normal, age-related levels, mean 26.6 (13.2) nmol/L, to levels indicative of severe GHD, 11.6 (6.7) nmol/L, (p <0.001). Only a small, non-significant decrease was noted in GH-treated patients. Full details are provided elsewhere [33].

Completion rates of both GWBI and W-BQ12 were 100% in this study. A significant between-group difference in change scores in W-BQ12 Energy was found [t(18) = 3.25, p = 0.004, 2-tailed] with scores of Placebo-group patients dropping from 6.83 (3.64) at baseline to 5.9 (4.12) at end-point, indicating reduced energy, while GH-treated patients' scores increased from 7.06 (2.08) to 8.13 (1.25) over this period. GWBI Total showed no significant change (p = 0.24).

## Discussion

The two studies described here have made a useful contribution towards the psychometric validation of two well-being questionnaires for use in adult growth hormone deficiency: the General Well-being Index and the 12-item version of the Well-being Questionnaire.

Despite only minor changes made to a few words and to item order when adapting the Psychological General Well-being Index for use in Britain [19], a forced 6-factor analysis of the British GWBI did not find the 22 items factoring out separately into PGWB subscales such as anxiety or self-control. Unforced factor analysis, however, produced three factors (albeit with substantial double loading), largely confirming the factors labelled as 'general mood/affect', 'life satisfaction/vitality', 'poor physical health/somatic complaints' in the earlier study by Gaston and Vogl, conducted with a non-clinical sample [22]. GWBI Factor 1 items (present study) covered several aspects of affect, and positive and negative well-being did not factor out separately as in the W-BQ12. Vitality (GWBI) items loaded together with positive well-being on Factor 2, but with the W-BQ12, the value of having a separate energy subscale was demonstrated in that W-BQ12 Energy was the only scale sensitive to change in the GH-Withdrawal study. Although the GWBI has a weak physical health factor, accounting for just 11% or the variance, this reflects the fact that one of the six subscales of the original PGWB concerned general health. However, the GWBI could not be described as a measure of health status as only a small proportion of items (3/22) concern physical health perceptions.

The internal consistency reliability of the whole GWBI scale was very high (>0.95) indicating that there may well be redundancy of items, particularly as two pairs of items are similarly worded, adding unnecessarily to respondent burden. The two general health items loading on Factor 3 in the unforced factor analysis had similar wording, and one or the other would appear to be redundant (if either were deleted from the scale then only two factors emerged in the unforced analysis). Although the reliability of the three potential GWBI subscales was high, overlap is considerable and we agree with the recommendation by McKenna et al [20] that there are no clear subscales to the questionnaire, and only a total score should be calculated.

Factor analysis of the W-BQ12 indicated three relatively clean factors providing evidence for W-BQ12 subscales of Negative Well-being and Positive Well-being and, although two W-BQ12 Energy items double loaded, it was possible to interpret the third W-BQ12 factor as an Energy subscale. There was support for the calculation of a total score for the questionnaire. The internal consistency reliability of subscales and W-BQ12 General Well-being combined scale was excellent. This high value would not indicate item redundancy, however, (as in the case of the GWBI), because the W-BQ12 is considerably shorter than the GWBI and none of the items are similar to each other (as in the GWBI).

Both well-being questionnaires had very high completion rates indicating good acceptability to patients, an indication of face validity. The strong correlations of both questionnaires with appropriate scales of the SF-36 (Mental Health and Vitality) but lower correlations with SF-36 Physical Functioning gave support for the concurrent validity of both questionnaires. The moderate correlations with Physical Functioning, however, indicate that well-being is associated with some aspects of health status.

There was preliminary evidence for construct validity in both GWBI and W-BQ12, although no prior hypotheses were formulated. Both questionnaires found significant differences between men and women, with women having generally lower well-being than men. It is a common finding that, in the general population, women have reduced well-being compared with men [43,44], although not all studies have found this [21]. Women with GHD have also tended to exhibit lower levels of well-being than men [10]. Both questionnaires showed correlations indicative of improving well-being for GH-treated patients with longer periods of GH treatment, as seen in previous research [17]. However, neither questionnaire found significant GH treatment-group differences either because the questionnaires were insufficiently sensitive to treatment-group differences, or there were no real differences in well-being between the two groups, possibly as a result of the fact that symptomatic patients were more likely to have been selected for treatment by the doctors in the clinics. Indeed none of the other questionnaires used in the study found GH-treated patients to have significantly better patient-reported outcomes (including health status and condition-specific quality of life) than non-treated patients [34]. It is possible, but perhaps more unlikely, that those receiving GH were not all adhering to the injection regimen (the patients' Insulin-like Growth Factor-I data at the time of the study were not collected).

The small sample size in the GH-Withdrawal study resulted in low power of analysis but, as anticipated, W-BQ12 Energy found a significant between-treatment-group difference in change scores over the withdrawal period, with reduced energy in placebo-treated patients at end-point. This provided a very preliminary indication of the W-BQ12's sensitivity to change, (there was no significant finding for the GWBI). This is in line with results of a study in which GH was discontinued in young adults with GHD [45] where a significant increase in psychological complaints (on the Hopkins Symptom Checklist [46]) was found after 6 months' discontinuation, although not across 12 months' discontinuation. It is possible that the 3-month withdrawal period of the present study was not long enough for significant change to be registered by the GWBI. All the previous studies of GH-replacement therapy that found significant improvement in psychological well-being using the PGWB, had a minimum 6 months' duration [13,14,17]. Further work on sensitivity to change with this patient group would be valuable in a larger and longer-term longitudinal intervention study, where GH treatment is being offered, not withdrawn.

The American PGWB has been the most widely used measure of psychological well-being in adult GHD, its six subscales sensitive to change in several studies of GH replacement. The British GWBI, on the hand, has no subscales, and the present study has confirmed that none can be recommended. This could be a disadvantage in this hormonal condition where low energy, increased anxiety and depression are key psychological aspects. Therefore, on the evidence provided by the present studies, the W-BQ12 can be recommended in preference to the GWBI to measure psychological well-being in adult GHD because the W-BQ12 has:

• excellent reliability, both of the whole scale and of the three subscales, with no indication of item redundancy;

• a clear factor structure supporting the use of subscales of Negative Well-being, Energy and Positive Well-being;

• fewer items (12 compared with 22 in the GWBI) and shorter response options (these are relatively long and vary from item to item in the GWBI, causing greater respondent burden);

• preliminary evidence of sensitivity to change.

To cover a wider range of patient-reported outcomes in adult GHD, it is recommended that the generic W-BQ12 be used in a battery of questionnaires that includes a generic measure of health status (e.g. the SF-36 [31]) and the HDQoL hormone deficiency-specific quality of life questionnaire [5].

## Conclusion

The W-BQ12 is recommended in preference to the GWBI to measure well-being in adult GHD as it is shorter, has useful subscales, has excellent internal consistency reliability with no indication of redundancy of items, and has preliminary indications of sensitivity to change.

## Competing interests

CM was funded by a research studentship from Lilly Industries Ltd, who also provided partial funding for JG. CB is copyright holder of the Well-being Questionnaire, Director of Health Psychology Research International and receives research grants and consultancies from many pharmaceutical companies.

## Authors' contributions

CM, CB and PS conceived and designed the cross-sectional study which was coordinated by CM who distributed the questionnaires. JG, DR-J and PS conceived and designed the controlled trial of GH withdrawal which was coordinated by JG who carried out biochemical measurements and distributed the questionnaires to participants. CM carried out the psychometric and statistical analyses of questionnaire data and drafted the manuscript. CB contributed to the interpretation of psychometric analyses and manuscript preparation. All authors read and approved the final manuscript.

## Copyright of W-BQ questionnaire

For access to and licence to use the W-BQ, contact the copyright holder, Clare Bradley PhD, Professor of Health Psychology, Health Psychology Research, Royal Holloway, University of London, Egham, Surrey, TW20 0EX. Email: c.bradley@rhul.ac.uk.
